# Ureteral calculi associated with high-altitude polycythemia: A case report

**DOI:** 10.1097/MD.0000000000024621

**Published:** 2021-02-19

**Authors:** Min Yang, Sen Cui, Tanna Wuren, Kexiong Ma, Ri-Li Ge, Linhua Ji

**Affiliations:** a Research Center for High Altitude Medicine, Qinghai University; b Key Laboratory of Application and Foundation for High Altitude Medicine Research in Qinghai Province; c Affiliated Hospital of Qinghai University, Xining; d Affiliated Huadu Hospital, Southern Medical University; e The Third School of Clinical Medicine, Southern Medical University, Guangzhou, China.

**Keywords:** case report, high-altitude polycythemia, hyperuricemia, ureteral calculus

## Abstract

**Rationale:**

High-altitude polycythemia (HAPC) is a common disease in high-altitude areas characterized by excessive erythrocyte proliferation and severe hypoxemia. Recently, the incidence of ureteral calculi has risen. However, cases of ureteral calculi associated with HAPC have not been reported.

**Patient concerns:**

We present the cases of 2 patients (26-year-old female, Case 1; 31-year-old male, Case 2) with HAPC who were born in the lowlands and worked in areas of high altitudes. Both patients were admitted to the hospital with acute severe pain in the ureter as the first symptom.

**Diagnoses:**

Urological examinations confirmed the presence of a ureteral stone. Interestingly, the biochemical tests showed elevated serum uric acid levels, and the calculous component analysis suggested anhydrous uric acid.

**Interventions:**

In the first case, the patient underwent extracorporeal shock wave lithotripsy. In the second case, the patient underwent right ureteroscopy and right ureteral stenting. The patient received postoperative anti-inflammatory, hemostatic, and rehydration therapy.

**Outcomes:**

Both patients recovered well with no recurrences observed upon regular re-examinations.

**Lessons:**

Recently, extensive research has demonstrated a significant correlation between hyperuricemia and HAPC. Therefore, we speculated that the occurrence of ureteral calculi among immigrants to the plateau might be related to hyperuricemia associated with HAPC. This case report and literature review highlights that the prevention of ureteral calculi in patients with polycythemia who immigrate to the plateaus from high-altitude areas should be considered. Additionally, the serum uric acid levels and urine pH should be monitored regularly.

## 1 Introduction

High-altitude polycythemia (HAPC) is a clinical syndrome caused by the inefficient acclimatization to high-altitude hypoxic environments. It is characterized by excessive red blood cell proliferation and severe hypoxemia.^[1]^ Recently, extensive research has demonstrated a significant correlation between hyperuricemia and HAPC, as hyperuricemia is a common complication in patients with HAPC.^[2–4]^ Uric acid is deposited in the kidneys in the form of sodium urate crystals when its concentration is supersaturated in the bloodstream. Furthermore, a persistent low urine pH similarly plays a key role in the pathogenesis of urolithiasis.^[5]^ Under high-altitude hypoxia, uric acid and other acidic metabolites increase in patients with HAPC, leading to the precipitation of uric acid crystals and subsequent deposition in the kidneys. Once the ureteral calculus is formed in such patients, the renal excretory function is aggravated, leading to the enormous accumulation of uric acid, thereby continuing a vicious circle. As medical assistance in the plateau areas is often limited, when patients with ureteral calculi do not receive timely treatment, a series of irreversible consequences may result. Therefore, the prevention of ureteral stones should be considered in patients with polycythemia after migrating to the plateaus. However, no studies on ureteral calculi concurrent with HAPC have been reported. Therefore, we sought to present 2 cases of ureteral calculi in patients born in lowlands and who worked in high-altitudes areas. We aimed to expand the literature on this disease and help clinicians consider methods to prevent its development.

## 2 Case presentation

### 2.1 Case 1

**Figure 1:**
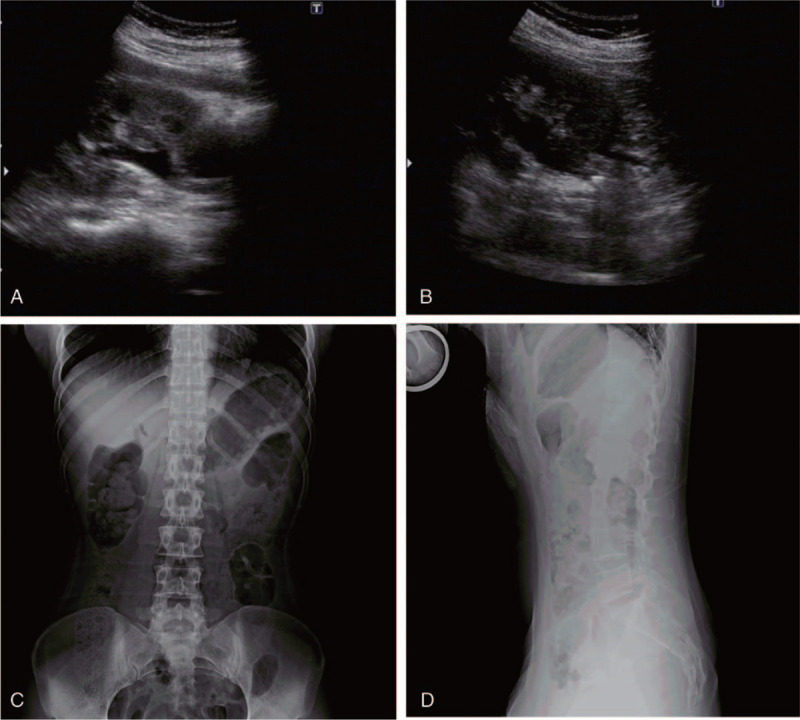
The urological Doppler ultrasound and radiograph of abdominal horizontal position radiograph (KUB) of patient 1. A and B, The urological Doppler ultrasound revealed hydronephrosis of the right kidney and right proximal ureteral calculus. C and D, The abdominal horizontal position radiograph in the right upper segment of the ureter deformable area showed high-density shadow because of the calculi.

The first case was of a 26-year-old Chinese woman who was admitted to a hospital (located at 4300 m; Maduo county, China) with complaints of severe pain in the right lower abdomen. The pain was accompanied by nausea, vomiting, chest tightness, and shortness of breath. The patient was a PhD student who was born in a lowland area (1100 m; Tianshui, China) and had moved to Maduo county 2 months earlier to conduct her scientific research. At that high altitude, she gradually developed shortness of breath, nocturnal sleep disturbance, and lip cyanosis. A clinical assessment revealed that she had developed tachycardia (pulse rate, 132/min), decreased SpO_2_ (73%), and hypotension (blood pressure, 90/58 mm of Hg). Due to the limited medical assistance in the area, she was immediately referred to our hospital for further evaluation and treatment. A physical examination of the upper abdomen showed tenderness and percussion pain over the right renal region and the ureter. A urological ultrasound showed that the spot separation of the right renal collecting system was 12 mm (Fig. 1A, B). On the right upper segment of the ureter, approximately 20 mm away from the renal pelvis, a bright spot with a diameter of approximately 7 mm was detected along with an acoustic shadow. The aforementioned findings revealed right hydronephrosis and right upper ureteral calculi. An abdominal horizontal position radiograph (KUB) showed a deformable area with a high-density shadow on the right upper segment of the ureter, suggesting the presence of a calculi (Fig. 1C, D). A routine blood investigation showed an increased absolute erythrocyte count and hemoglobin level (6.58 × 10^12^/L and 192 g/L, respectively). Further clinical biochemical tests revealed a high uric acid level (458 μmol/L). Routine urine examination was positive for occult blood (2+) and ketone bodies (2+). The peripheral blood Janus kinase 2 (*JAK2*) gene test showed no significant abnormalities. After evaluation, the patient underwent extracorporeal shock wave lithotripsy. Analysis of stone components suggested anhydrous uric acid. Additionally, no recurrences were observed upon regular re-examinations at 1 year postoperatively.

### 2.2 Case 2

**Figure 2:**
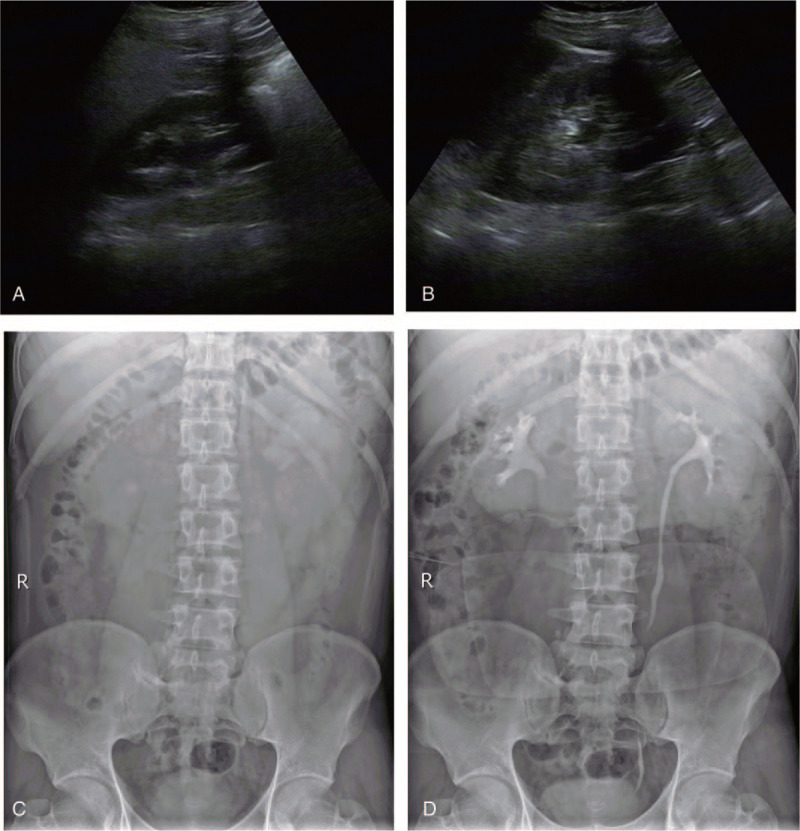
Ultrasonography of the urinary system, KUB, and intravenous urography (IVP) of patient 2. A, Right hydronephrosis. B, Right ureterectasia. C, KUB showed no positive urinary calculi in the bilateral renal area or ureteral path. D, IVP showed right hydronephrosis and dilatation in the upper and middle segments of the right ureter.

The second case was of a 31-year-old Chinese man who was admitted to our hospital with complaints of right lumbar pain accompanied by gross hematuria for 3 days. The patient was born in a lowland area (47 m; Yantai, China) and worked as an engineer in the highlands (2994 m; Haixi, China) for 3 years. He had a 2-year history of HAPC, without receiving any treatment. A physical examination revealed a right renal and ureteral tenderness, slight cyanosis of the lips, telangiectasia of the face, and conjunctival hyperemia. A clinical assessment revealed that he had tachycardia (pulse rate, 118/min) and reduced SpO_2_ (79%). The patient's body temperature, respiratory rate, and blood pressure were within the normal limits. Ultrasonography of the urinary system indicated an 11-mm separation of the right kidney collection system (Fig. 2A). The inner diameter of the upper part of the right ureter was 8 mm, while the middle and lower parts were not clearly displayed (Fig. 2B). The left ureter did not dilate, and KUB showed no positive urinary calculi in the bilateral renal area and ureteral path (Fig. 2C). Intravenous urography showed right hydronephrosis after intravenous contrast agent injection (Fig. 2D). Dilatation of the upper and middle segments of the right ureter was observed, whereas no dilatation, hydronephrosis, or damage was observed in the left ureter. From the imaging results, we diagnosed incomplete obstruction of the right middle and lower ureter. A routine blood investigation revealed increased absolute erythrocyte count and hemoglobin levels (7.78 × 10^12^/L and 225 g/L, respectively). Further clinical biochemical tests revealed high uric acid (579 μmol/L) and creatinine (64 μmol/L) levels. A routine urine examination was positive for occult blood (3+) and protein (1+). The peripheral blood *JAK2* gene test, tumor marker levels, and heart color Doppler ultrasound results showed no significant abnormalities. The patient underwent right ureteroscopy and right ureteral stenting. After the operation, the patient was administered anti-inflammatory, hemostatic, and rehydration therapy. Analysis of stone components suggested anhydrous uric acid. Afterward, the patient returned to the plateau to resume his previous work, and no recurrences were observed upon regular re-examinations at 3 months postoperatively.

## 3 Discussion and conclusions

In this report, we present, for the first time, 2 cases of patients with HAPC who were born at areas of low altitudes and developed ureteral calculi while residing at high-altitude areas. According to the chronic mountain sickness (CMS) Qinghai integral standard of the 6th Plateau Medicine Conference in 2004,^[6]^ the CMS integral of patients 1 and 2 was 8 and 12 points, respectively. Both cases presented with acute ureteral colic, as the first symptom, and increased serum uric acid levels. Considering the aforementioned data, we speculate that polycythemia may be a risk factor for ureteral calculi in individuals who migrate to the highlands. To the best of our knowledge, this is the first report presenting the occurrence of HAPC with ureteral calculi in highlands immigrants.

The etiology of ureteral calculi remains unknown and is related to geographical locations, climatic environments, dietary habits, occupations, and basic diseases.^[7]^ Ureteral calculi account for 65% of urinary calculi cases, and the incidence has been rising recently.^[8]^ In general, hyperuricemia significantly increases the incidence of urinary calculi.^[9]^ When the uric acid concentration is supersaturated, it gets deposited in the renal tissues in the form of sodium urate crystals. Researchers have found that there was a 75% incidence rate of urinary tract stones in patients with hyperuricemia caused by rare single-gene enzyme disorders.^[10]^

Uric acid is the end-product of purine nucleotide metabolism, and approximately two-thirds of uric acid is excreted through the urine.^[11]^ Hyperuricemia is a common condition in patients with HAPC, and multiple factors contribute to this process. Excessive erythrocyte proliferation and apoptosis of HAPC may contribute to increased uric acid levels.^[12]^ Similarly, hyperuricemia is commonly observed in patients with erythrocytosis secondary to lung diseases and polycythemia vera,^[13,14]^ which indirectly indicates the correlation between erythrocytosis and uric acid. Moreover, the filtration function of the kidneys can be inhibited by increased blood viscosity. In addition, high-altitude hypoxia increases the concentration of lactic acid, especially in patients with HAPC, which can competitively inhibit the excretion of uric acid.^[15]^

A high uric acid concentration and the persistent low pH of urine play a key role in the pathogenesis of uric acid calculi.^[5]^ Urine pH can affect the formation of different types of urinary stones. Under normal circumstances, urine pH is approximately 5.7 to 6.3. Consequently, a uric acid calculus is easily formed when the urine pH is <5.5, while calcium phosphate and struvite calculi readily form when the urine pH is high.^[16,17]^ In addition to pure uric acid stones, uric acid or sodium urate precipitation provides a nidus for the subsequent formation of calcium oxalate stones, thereby further contributing to the overall incidence of stones.^[18]^ Individuals are at risk of developing hyperventilation and respiratory alkalosis on exposure to acute hypoxia, although their pH is kept within a normal range.^[19]^ Moreover, the formation of urinary calculi has a relatively slow progression rate; hence, acute hypoxic exposure has a limited effect on urinary stone formation. However, for patients with HAPC who are exposed to chronic hypoxia, a high uric acid concentration plays an important role in the formation of uric acid stones. Moreover, the concentrations of uric acid metabolites in such patients are increased, and uric acid crystals are aptly precipitated and deposited in the kidneys. Urinary calculus is formed in patients with HAPC, which in turn aggravates the renal excretion function that can ultimately lead to uric acid formation, thereby continuing a vicious circle.

The prevention of ureteral calculi associated with HAPC is particularly important for several reasons. First, in routine blood investigations, the serum uric acid level and 24-h urinary pH should be regularly monitored in individuals who have migrated to high-altitude areas. Second, drinking a sufficient quantity of water is recommended because a significant amount of liquid intake has a protective effect on the incidence of urolithiasis. Third, the dietary structure should be appropriately adjusted by reducing the intake of foods with high purine content. Notably, type II diabetes, metabolic syndrome, and obesity are associated with hyperuricemia and are equally risk factors for uric acid stone formation.^[20]^ Therefore, the uric acid levels of people with these diseases migrating to the plateau should be monitored to reasonably prevent the occurrence of urolithiasis.

## 4 Conclusions

Urolithiasis associated with HAPC might be a public health concern worldwide. These cases highlight the importance of implementing a system to monitor serum uric acid levels in individuals residing in high-altitude areas, which could help prevent ureteral calculi associated with HAPC. Meanwhile, publicity and education on this matter should be strengthened to improve the dietary structure and develop the habit of drinking a sufficient quantity of water. Further research on the underlying mechanisms of urinary stone formation in patients with HAPC, including stone analysis and 24-h urine composition analysis, could provide useful information regarding the association between urinary stone formation and HAPC.

## Author contributions

LJ, SC, and MY designed the study. KM performed the literature search. SC and MY performed the medical records analysis and interpretation. MY and LJ drafted the manuscript and revised the main body of the manuscript. LJ, SC, RG, and TW revised the final manuscript, instructed the writing of the manuscript. All authors read and approved the final manuscript.

**Conceptualization:** Min Yang, Sen Cui, Linhua Ji.

**Investigation:** Kexiong Ma.

**Writing – original draft:** Min Yang.

**Writing – review & editing:** Tanna Wuren, Ri-Li Ge, Linhua Ji.

## References

[1] Wang ZK Liu FJ Ye SL. Plasma proteome profiling of high-altitude polycythemia using TMT-based quantitative proteomics approach. J Proteomics 2019;194:60–9. 30605725 10.1016/j.jprot.2018.12.031

[2] Li M Ci RCM. Changes of serum uric acid in patients with polycythemia at 4300m altitude. [original article in Mandarin]. Tibetan Med 2010;2:43–4.

[3] Wang SY Lou XM Li MY. Analysis of the risk factors of uricemia in men at high altitude. [original article in Mandarin]. J High Altitude Med 2014;04:32–4.

[4] Yu HT Ci RCM Jian XJ. Effect of hemoglobin concentration on serum uric acid level in physical examination population at high altitude. [original article in Mandarin]. Tibet Sci Technol 2018;12:47–8.

[5] Liu CJ Wu JS Huang HS. Decreased associated risk of gout in diabetes patients with uric acid urolithiasis. J Clin Med 2019;8:1536. 10.3390/jcm8101536 PMC6832126 31557790

[6] Leon-Velarde F Maggiorini M Reeves JT. Consensus statement on chronic and subacute high altitude diseases. High Alt Med Biol 2005;6:147–57. 16060849 10.1089/ham.2005.6.147

[7] Trinchieri A Montanari E. Prevalence of renal uric acid stones in the adult. Urolithiasis 2017;45:553–62. 28258472 10.1007/s00240-017-0962-5

[8] Corbo J Wang J. Kidney and ureteral stones. Emerg Med Clin North Am 2019;37:637–48. 31563199 10.1016/j.emc.2019.07.004

[9] Kong WY. A survey of the relationship between urolithiasis and hyperuricemia in lianshan area. [original article in Mandarin]. Practical Clin J Integr Traditional Chinese Western Med 2017;09:21–3.

[10] Moe OW Abate N Sakhaee K. Pathophysiology of uric acid nephrolithiasis. Endocrinol Metab Clin North Am 2002;31:895–914. 12474637 10.1016/s0889-8529(02)00032-4

[11] Pehlivanlar-Kucuk M Kucuk AO Ozturk CE. The association between serum uric acid level and prognosis in critically ill patients, uric acid as a prognosis predictor. Clin Lab 2018;64:1491–500. 30274009 10.7754/Clin.Lab.2018.180334

[12] Jefferson JA Escudero E Hurtado ME. Hyperuricemia, hypertension, and proteinuria associated with high-altitude polycythemia. Am J Kidney Dis 2002;39:1135–42. 12046023 10.1053/ajkd.2002.33380

[13] Chen M Shen Q Gu J. Hyperuricemia secondary to polycythemia vera. Hematology branch of Chinese Medical Association. [original article in Mandarin]. Papers of the Eighth National Hematology Academic Conference of Chinese Medical Association 2004;244.

[14] Sunnetcioglu A Gunbatar H Yildiz H. Red cell distribution width and uric acid in patients with obstructive sleep apnea. Clin Respir J 2018;12:1046–52. 28296212 10.1111/crj.12626

[15] Ba YG Zhang RX Qin F. Study on the relationship between high altitude polycythemia and renal damage. [original article in Mandarin]. J High Alt Med 2017;01:15–8.

[16] Das P Gupta G Velu V. Formation of struvite urinary stones and approaches towards the inhibition—a review. Biomed Pharmacother 2017;96:361–70. 29028588 10.1016/j.biopha.2017.10.015

[17] Ennis JL Asplin JR. The role of the 24-h urine collection in the management of nephrolithiasis. Int J Surg 2016;36:633–7. 27840312 10.1016/j.ijsu.2016.11.020

[18] Maalouf NM Cameron MA Moe OW. Novel insights into the pathogenesis of uric acid nephrolithiasis. Curr Opin Nephrol Hypertens 2004;13:181–9. 15202612 10.1097/00041552-200403000-00006

[19] Sato M Severinghaus JW Powell FL. Augmented hypoxic ventilatory response in men at altitude. J Appl Physiol (1985) 1992;73:101–7. 1506356 10.1152/jappl.1992.73.1.101

[20] Khan SR Pearle MS Robertson WG. Kidney stones. Nat Rev Dis Primers 2016;2:16008. 27188687 10.1038/nrdp.2016.8 PMC5685519

